# Characteristics of chronic thromboembolic pulmonary hypertension in Ireland

**DOI:** 10.1177/20458940211048703

**Published:** 2021-10-08

**Authors:** Sarah Cullivan, Ciara McCormack, Marissa O’Callaghan, Barry Kevane, Fionnuala NiAinle, Brian McCullagh, Sean P. Gaine

**Affiliations:** 1 National Pulmonary Hypertension Unit, 8881 Mater Misericordiae University Hospital, Dublin, Ireland; 2 Department of Haematology, 8881 Mater Misericordiae University Hospital, Dublin, Ireland

**Keywords:** chronic thromboembolic pulmonary hypertension, pulmonary endarterectomy, balloon pulmonary angioplasty

## Abstract

Chronic thromboembolic pulmonary hypertension (CTEPH) is a rare and under-recognised complication of acute pulmonary embolism. Information regarding the characteristics of CTEPH in Ireland is limited, and the aim of this retrospective cohort study was to address this knowledge gap. Seventy-two cases of CTEPH were diagnosed in the National Pulmonary Hypertension Unit (NPHU) in Ireland between 2010 and 2020. This accounted for 6% of all referrals to the unit and translates to an estimated annual incidence of 1.39 per million population (95% confidence interval, 0.33–2.46). The prevalence of diagnosed CTEPH in Ireland in 2020 was estimated at 12.05 per million population (95% CI 9.00–15.10). The average duration of symptoms prior to CTEPH diagnosis was 23 (±22) months. Patients with CTEPH were more likely to be male (n = 40, 56%), older (60 ± 17 years) and have identifiable risk factors for CTEPH (n = 61, 85%) at diagnosis. Regarding treatment, pulmonary hypertension (PH) vasodilator therapy was prescribed in 75% (n = 54) within 12 months of diagnosis, inferior vena cava filters were placed in 24% (n = 17) and 97% (n = 70) of cases were anticoagulated. Pulmonary endarterectomy was performed in 35% (n = 25), balloon pulmonary angioplasty in 6% (n = 4). One-, three- and five-year survival was 93%, 80% and 65% from the time of diagnosis, and this was significantly better in patients who underwent pulmonary endarterectomy (p = 0.01). This is the first study describing the characteristics of CTEPH in Ireland and highlights suboptimal disease recognition and referral for the assessment for pulmonary endarterectomy.

## Introduction

CTEPH is a rare and under-recognised disease of the pulmonary vasculature, that is classified within WHO Group 4 pulmonary hypertension (PH). It is characterised by fibrotic intravascular occlusions within the pulmonary arterial tree and a secondary microvasculopathy of smaller vessels that are exposed to increased flow and shear stress. Pulmonary capillaries and veins are also implicated through exposure to systemic pressures via hypertrophied collateral bronchial arteries.^1^^–3^ While numerous risk factors and predisposing conditions for this disease have been identified, the epidemiology and pathobiology remain incompletely defined, and reliable biomarkers of thrombus transformation are lacking.^3^ The annual incidence of CTEPH is estimated between 3.1 and 6.0 cases per million population, and prevalence between 25.8 and 38.4 cases per million population, though there is considerable heterogeneity in the reported figures.^4^ Disease under-recognition and diagnostic delays are persistent problems, with important clinical consequences.

The diagnosis of CTEPH requires right heart catheterisation (RHC) to confirm PH. Imaging typically consists of a combination of ventilation perfusion (V/Q) scintigraphy, computed tomography pulmonary angiography (CTPA) and invasive pulmonary angiogram, to demonstrate mismatched perfusion defects and to define the distribution of intravascular disease. Lifelong anticoagulation is recommended in all cases, and pulmonary endarterectomy (PEA) is the treatment of choice for operable disease, due to established symptomatic and survival benefits.^1^^–3^ Treatment options for inoperable disease include targeted PH therapy and BPA, while lung transplantation is considered in advanced disease in selected cases.^3^

There is a paucity of published data regarding the characteristics of CTEPH in Ireland and therefore we sought to address this knowledge gap, with a specific focus on the incidence, treatment patterns and survival of patients with CTEPH in Ireland.

## Methods

This retrospective cohort study complied with the declaration of Helsinki and received ethical approval from the institutional ethical review board (IRB:1/378/2176TMR).

Data regarding individual patients referred to the National Pulmonary Hypertension Unit (NPHU) between January 2010 and December 2020 was collected retrospectively and fully anonymised. Confirmed cases of CTEPH, diagnosed by right heart catheterisation (RHC) and defined by a mean pulmonary artery pressure (mPAP) greater than 20 mmHg and pulmonary vascular resistance greater than 3 wood units were selected for further analysis.^3^ Data regarding patient and treatment characteristics and cumulative survival were collected from hospital paper charts and the electronic IT system (PatientCentre). The annual incidence of CTEPH during the study period was calculated using population estimates provided by the central statistics office for the Republic of Ireland and the 2016 census.^5^

Statistical analysis was performed using GraphPad online statistical software. Continuous variables were expressed as mean ± standard deviation and categorical variables as n (%). An unpaired t-test was used to calculate significance between means and Fisher’s exact test was used to determine associations between categorical variables. Survival estimates were made using the Kaplan-Meier method, with comparisons performed by the log-rank test. A value less than 0.05 was considered statistically significant (p < 0.05).

## Results

### Study population

Of the 1243 referrals to the NPHU between 2010 and 2020, 72 cases of CTEPH were diagnosed. This accounted for 6% of all referrals and 14% of confirmed PH cases during that period. This translates to a calculated annual incidence of 1.39 per million population (95% CI 0.33–2.46). This varied each year, with 1.34 cases per million inhabitants identified in 2010 (95% CI 0.27–2.42), which increased to 1.81 per million population in 2020 (95% CI 0.63–2.99). The estimated prevalence of CTEPH in Ireland in 2020 was 12.05 per million population (95% CI 9.00–15.10).

At diagnosis, patients with CTEPH were typically older, with a mean age of 60 ± 17 years, and a slight male preponderance at 56% (n = 40). The mean time from symptom onset to diagnosis was 23 (±22) months. Ninety nine percent (n = 71) reported dyspnoea and 61% (n = 44) reported WHO functional class (FC) III symptoms. At diagnosis, the mean b-type natriuretic peptide (BNP) was elevated at 274 ng/L (±324), the six minute walk distance (6MWD) 339 m (±144) and the mean pulmonary artery pressure (mPAP) was 42 ± 10 mmHg. Risk factors for CTEPH were identified in 85% (n = 61) and are displayed in Table 1.

**Table 1.** The baseline characteristics of patients diagnosed with CTEPH.

Baseline characteristics	
Patients, n	72
Sex: male n (%)	40 (56)
Age (years): mean ± SD	60 ± 17
Duration of symptoms (months): mean ± SD	23 ± 22
WHO functional class (FC), % I/II/III/IV	1/31/61/7
BNP (ng/L): mean ± SD	274 ± 324
6-minute walk distance (meters): mean ± SD	339 ± 144
Risk stratification (ESC/ERS): n (%)	
Low risk	23 (32)
Intermediate risk	30 (42)
High risk	12 (17)
Identifiable risk factors for CTEPH: n (%)	61 (85)
Prior history of PE	50 (69)
History of cancer	11 (15)
Chronic infected lines/PPM	2 (3)
Chronic osteomyelitis	3 (4)
Inflammatory bowel disease	3 (4)
Hypothyroidism, prescribed thyroid hormone replacement	8 (11)
Splenectomy	4 (6)
Thrombophilia	4 (6)
Myeloproliferative disorders	1 (1)
Right heart catheterisation	
mRAP (mmHg)	9 ± 5
mPAP (mmHg)	42 ± 10
PAWP (mmHg)	11 ± 4
CO (L/min)	4 ± 1
PVR (WU)	8 ± 4
CTEPH distribution	
Proximal disease	47 (65)
Distal disease	25 (35)

CTEPH: chronic thromboembolic pulmonary hypertension; SD: standard deviation; WHO: World Health Organisation; BNP: B-type natriuretic peptide; ESC: European Society of Cardiology; ERS: European Respiratory Society; VTE: venous thromboembolism; PPM: permanent pacemaker; mRAP: mean right atrial pressure; mPAP: mean pulmonary artery pressure; PAWP: pulmonary artery wedge pressure; CO: cardiac output; PVR: pulmonary vascular resistance; WU: Wood units.

Data regarding duration of symptoms pre diagnosis, BNP, 6MWD, risk stratification and haemodynamic parameters were incomplete. Information regarding the duration of symptoms pre diagnosis was available in 82% (n = 59), BNP in 94% (n = 68), 6MWD in 57% (n = 41) and risk stratification in 90% (n = 65). Mean right atrial pressure (mRAP) was available in 68% (n = 49) cases, mPAP in 94% (n = 55), pulmonary arterial wedge pressure (PAWP) in 76% (n = 55), cardiac output (CO) in 64% (n = 46) and pulmonary vascular resistance (PVR) in 54% (n = 39).

### Treatment

Anticoagulation was prescribed in 97% (n = 70), with warfarin in 58% (n = 42), apixaban in 11% (n = 8), rivaroxaban in 22% (n = 16) and low molecular weight heparin in 6% (n = 4). In the remaining two cases, anticoagulation was contraindicated in one case and declined in a further case. Inferior vena cava (IVC) filters were inserted at the time of CTEPH diagnosis in (24%, n=17), all of which occurred between the years 2010 and 2015.

Seventy five percent (n = 54) of cases were referred to the specialist CTEPH multidisciplinary team (MDT) in Papworth. PH specific therapy was prescribed in 75% (n = 54) during the first 12 months following diagnosis. This consisted of monotherapy in 53% (n = 38), double combination therapy in 21% (n = 15) and triple combination therapy in 1% (n = 1). Seventy-five percent (n = 40) of these prescriptions were off-label.

CTEPH distribution was defined as proximal disease in 65% (n = 47) and distal in 35% (n = 25) (Supplementary figures 1 and 2). PEA was performed in 35% (n = 25) and BPA in 6% (n = 4). Of note, one individual underwent both procedures. The average number of BPA sessions in these four cases was 3.3 ± 1.5. In the remaining cases, CTEPH was considered in-operable and not amenable to angioplasty in 15% (n = 11) due to anatomical characteristics, intervention was contraindicated due to comorbidities in 15% (n = 11) and declined by individual patients in 8% (n = 6). Seven percent (n = 5) died before final treatment decisions were made, one patient was lost to follow-up. Decisions are pending in 14% (n = 10) due to delays associated with the COVID19 pandemic. Additional treatment characteristics are highlighted in Table 2.

**Table 2.** The treatment characteristics of patients with CTEPH.

Treatment characteristics	
Anticoagulation: n (%)	
Warfarin	42 (58)
Apixaban	8 (11)
Rivaroxaban	16 (22)
Low molecular weight heparin	6 (4)
IVC filter: n (%)	17 (24)
PH therapy: n (%)	54 (75)
Single-agent therapy	38 (53)
PD5 inhibitor	9 (24)
sGCS	13 (35)
ERA	16 (43)
Double combination therapy	15 (21)
PD5 inhibitor & ERA	8 (53)
sGCS + ERA	6 (40)
ERA + PGI_2_	1 (7)
Triple combination therapy	1 (1)
PD5 inhibitor + ERA + PGI_2_	1 (100)
Surgery & Interventional procedures: n (%)	29 (40)
Pulmonary endarterectomy	25 (35)
Balloon pulmonary angioplasty	4 (6)

IVC: inferior vena cava; PH: Pulmonary Hypertension; PD5: phosphodiesterase type 5; sGCS: soluble guanylate cyclase stimulator; ERA: endothelin receptor antagonist; PGI_2_: prostacyclin; PEA: pulmonary endarterectomy; BPA: balloon pulmonary angioplasty.

### Survival

**Fig. 1.**
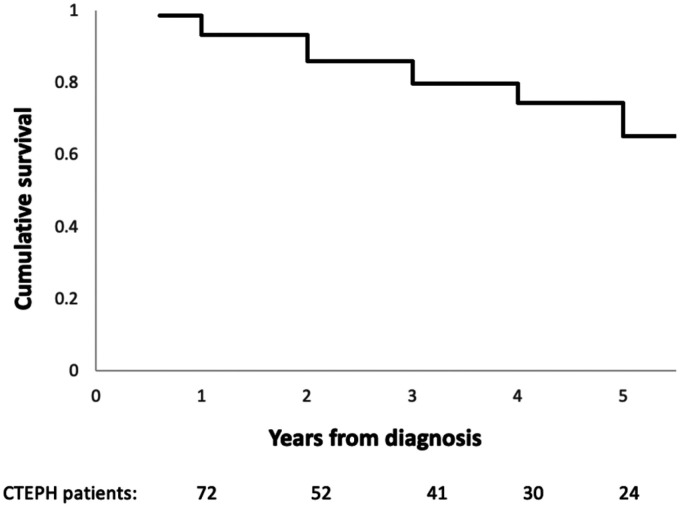
Kaplan Meier graph illustrating the cumulative survival of patients diagnosed with CTEPH in Ireland between 2010 and 2020.

**Fig. 2.**
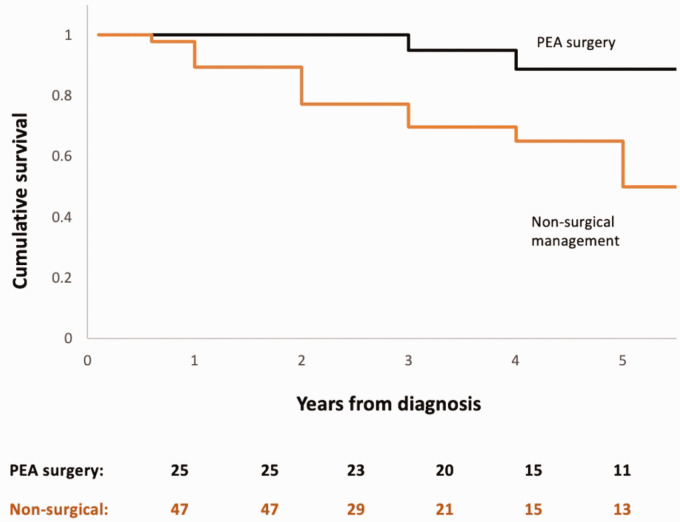
Kaplan Meier graph displaying the cumulative survival of patients treated with PEA and patients who did not undergo surgical intervention. Log rank test, p = 0.01.

The one-, three- and five-year survival was 93%, 80% and 65% from the date of CTEPH diagnosis (Fig. 1). Patients who died (n = 19, 26%) had a higher pulmonary vascular resistance (PVR) at diagnosis (p = 0.05) and significantly lower rates of surgical intervention with PEA (p = 0.02). PEA was associated with a significant difference in survival, as the 1, 3 and 5 year survival following PEA was 100%, 95% and 89%, while this was 90%, 70% and 50% for patients who did not undergo surgical intervention (p = 0.01) (Fig. 2).

## Discussion

CTEPH is a rare disease of the pulmonary vasculature, which is associated with considerable morbidity and mortality.^1^,^2^ This study explores the characteristics of CTEPH in Ireland and highlights important aspects of disease incidence, treatment patterns and survival in this population.

We report an estimated annual incidence of CTEPH in Ireland of 1.39 cases per million population and a prevalence of 12.05 cases per million population between 2010 and 2020. While the true global incidence of CTEPH is unknown, with reports varying from 0.9 to 39 cases per million population, these figures suggest that CTEPH may be underrecognised in Ireland.^4^ A systematic review of national registry data from the UK, Sweden and Latvia demonstrated that the incidence of CTEPH is probably closer to 3.1–6.0 per million population.^4^ In fact, suboptimal CTEPH diagnosis is not unique to our cohort, but rather reflects a global problem. An epidemiological analysis of CTEPH in Europe, the USA and Japan projected that only 16% of CTEPH cases would be identified in 2015, and that many of these individuals would present with advanced disease.^6^

Unfortunately the diagnosis of CTEPH is often delayed, as exemplified in our study. Patients reported an average duration of symptoms of 23 months prior to diagnosis. This is consistent with existing registry data and potentially reflects a combination of suboptimal patient and physician disease awareness. The non-specific nature of CTEPH symptoms and overlapping features with alternative cardiorespiratory conditions provide additional barriers to early disease recognition.^7^^–10^ Delayed diagnosis has meaningful clinical implications, as CTEPH is amenable to specific medical, interventional and surgical therapies that have been shown to improve clinical outcomes. Moreover, diagnostic delays have been correlated with worse haemodynamic profiles and reduced survival.^11^ The anatomical distribution of CTEPH was proximal disease in 65% of cases in this study. These results were unsurprising, as CT imaging is often performed early in the diagnostic pathway of patients with undefined dyspnoea and proximal disease is more readily identified on CT when compared to distal CTEPH.

CTEPH is considered a complication of pulmonary embolism (PE) and a history of acute PE is observed in 50–75% of cases.^3^,^9^,^12^ Risk factors for CTEPH were identified in 85% of patients and 69% reported a history of PE in our cohort. Patients diagnosed with CTEPH in this study were typically older and there was no female predominance, which is consistent with previous reports. Treatment comprised of combinations of anticoagulation, IVC filters, targeted PH therapy, BPA and PEA. Anticoagulation consisted predominantly of the vitamin K antagonist (VKA) warfarin (n = 42, 58%) and novel oral anticoagulants (NOACs) (n = 24, 33%). While VKAs have been the mainstay of anticoagulation in CTEPH,^3^ NOACs are increasingly used in clinical practice for this indication. Available data regarding the safety and efficacy of NOACs in CTEPH provide inconsistent and conflicting messages. Some studies report significantly higher rates of venous thromboembolism^13^ and fresh thrombus at PEA^14^ in individuals prescribed NOACs when compared to VKA users. However, other studies, including the multicentre international EXPERT registry, suggest that bleeding and thrombotic complications are equivalent between these agents.^15^

Until recently, IVC filter placement in patients with CTEPH was common practice.^16^^–18^ This was guided by early evidence suggesting a potential role in the prevention of recurrent PE^19^ and the requirement for repeat PEA.^20^ Their routine use has now fallen out of vogue, due to a paucity of high-quality studies and the absence of evidence of a survival benefit.^21^,^22^ No IVC filters have been placed in our centre for this indication since 2015, though they were frequently placed prior to this (24%, n=17).

The NPHU in Ireland was established in 2003 and is one of nine specialised PH centres in Ireland and the UK with access to the centralised PEA and BPA program in Papworth in the UK.^18^ Reciprocal healthcare between Ireland and the UK is enabled by the common travel area, which is independent of and predates European Union membership. This provides a framework to support publicly funded health services in each state, including PEA and BPA. Typically patients with suspected CTEPH are assessed and managed locally, and subsequently referred to the specialised CTEPH MDT in Papworth for an expert opinion regarding suitability for PEA and BPA. This study highlights suboptimal referrals for same at 75%.

PEA is considered the gold standard for operable CTEPH due to established symptomatic and prognostic benefits and therefore it is recommended that all patients with CTEPH are considered for this procedure.^18^ There are few absolute contraindications to PEA and typically at least two-thirds of cases are technically operable. However, not all patients with operable disease undergo PEA, for individual personal or medical reasons.^3^,^7^,^8^,^18^,^23^,^24^ Only 51% (n = 24) of patients with proximal disease (and 35% of the entire cohort) underwent PEA between 2010 and 2020, which is considerably lower than other centres. Patients who underwent PEA were significantly younger than patients who did not (p = 0.0065). It is possible that travel to the UK for this intervention may impact treatment decisions for some older patients with comorbidities. This may result in a potential overdependence on medical therapy. Interestingly, 75% (n = 54) of patients in our centre were prescribed PH specific therapy in the first 12 months following CTEPH diagnosis. An international physician survey of CTEPH management previously highlighted this concerning trend, as many physicians only consider PEA if medical therapy fails.^25^ Furthermore, patients may decline intervention due to perceived operative risks, reinforcing the importance of patient education and informed, shared decision-making.^26^ There were no perioperative deaths in the cohort that underwent PEA and none of the patients in this study underwent lung transplantation during this 11-year period. Patient survival in this study was comparable to other centres and significantly improved in those who underwent PEA, in keeping with previously published outcomes.^7^,^8^ Of note, BPA may significantly improve survival in the non-surgical group in the future. Decisions regarding operability are pending in 14% (n = 10) due to significant service disruptions associated with the COVID19 pandemic.

Limitations of this study include its retrospective and descriptive nature. Nonetheless, it provides valuable information which can inform the PH community and countries with similar healthcare arrangements. We have identified an annual incidence of CTEPH that is lower than anticipated and suggests suboptimal disease recognition. This requires a national strategy to improve disease awareness and subsequent referral of suspected cases. The role of screening high risk individuals post PE with specific risk factors and predisposing conditions is a subject of significant interest and ongoing debate, and requires further research.^3^ While routine echocardiography in all PE survivors is not recommended,^12^ potential screening tools that have been explored include clinical prediction scores such as the ‘CTEPH predication score’, that incorporates seven clinical variables to estimate the pre-test probability of CTEPH post-acute PE.^27^,^28^ Standardised reading of CTPA scans for radiological parameters of CTEPH at the time of acute PE may also assist case identification, as highlighted by the InShape III study.^29^ Finally, this study has highlighted a possible overreliance on medical therapy and suboptimal referral to the centralised CTEPH MDT.

## Conclusion

This study provides valuable information regarding the epidemiology, treatment characteristics and survival of patients with CTEPH in Ireland, and suggests that CTEPH is potentially under-recognised, with less surgical intervention than expected.

## Key message

This study addresses the paucity of data regarding the characteristics of CTEPH in Ireland and provides valuable information regarding the epidemiology, treatment characteristics and survival of this cohort.

## Supplemental Material

sj-pdf-1-pul-10.1177_20458940211048703 - Supplemental material for Characteristics of chronic thromboembolic pulmonary hypertension in Ireland Click here for additional data file. Supplemental material, sj-pdf-1-pul-10.1177_20458940211048703 for Characteristics of chronic thromboembolic pulmonary hypertension in Ireland by Sarah Cullivan, Ciara McCormack, Marissa O’Callaghan, Barry Kevane, Fionnuala NiAinle, Brian McCullagh and Sean P. Gaine in Pulmonary Circulation

sj-pdf-2-pul-10.1177_20458940211048703 - Supplemental material for Characteristics of chronic thromboembolic pulmonary hypertension in Ireland Click here for additional data file. Supplemental material, sj-pdf-2-pul-10.1177_20458940211048703 for Characteristics of chronic thromboembolic pulmonary hypertension in Ireland by Sarah Cullivan, Ciara McCormack, Marissa O’Callaghan, Barry Kevane, Fionnuala NiAinle, Brian McCullagh and Sean P. Gaine in Pulmonary Circulation
