# Supplementary figures and images for: Characteristics of chronic thromboembolic pulmonary hypertension in Ireland
Source: Pulm Circ. 2021 Oct 8;11(4):20458940211048703. doi: 10.1177/20458940211048703 (PMC8504238; doi:10.1177/20458940211048703)

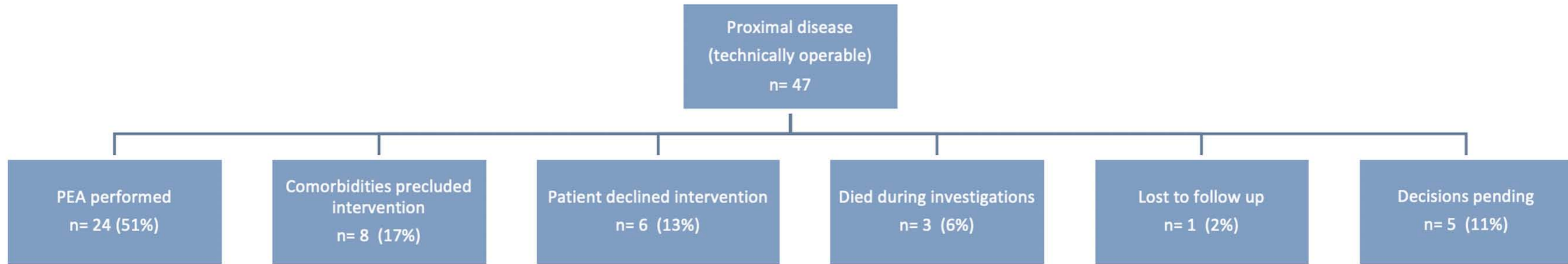

Supplement: sj-pdf-1-pul-10.1177_20458940211048703 - Supplemental material for Characteristics of chronic thromboembolic pulmonary hypertension in Ireland [file sj-pdf-1-pul-10.1177_20458940211048703.pdf]
