# Supplementary material for: Characteristics of chronic thromboembolic pulmonary hypertension in Ireland
Source: Pulm Circ. 2021 Oct 8;11(4):20458940211048703. doi: 10.1177/20458940211048703 (PMC8504238; doi:10.1177/20458940211048703)
Supplement: sj-pdf-2-pul-10.1177_20458940211048703 - Supplemental material for Characteristics of chronic thromboembolic pulmonary hypertension in Ireland [file sj-pdf-2-pul-10.1177_20458940211048703.pdf]

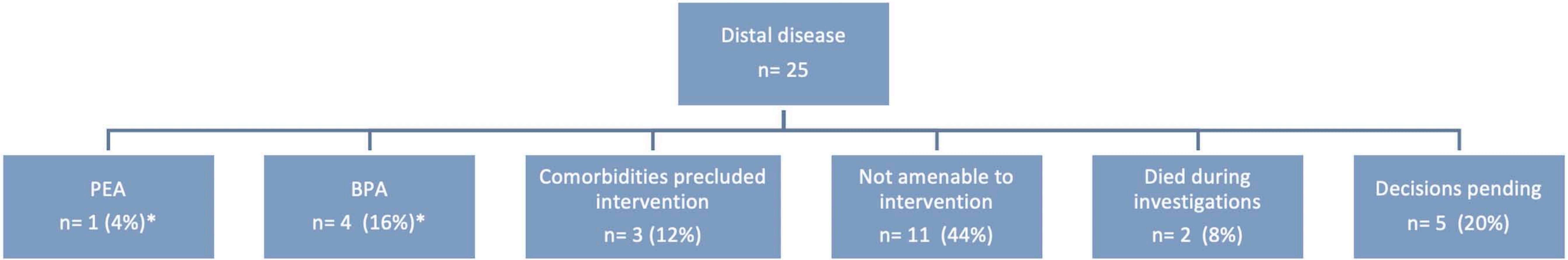
Distal disease
n= 25
PEA
n= 1 (4%)*
BPA
n= 4 (16%)*
Comorbidities precluded intervention
n= 3 (12%)
Not amenable to intervention
n= 11 (44%)
Died during investigations
n= 2 (8%)
Decisions pending
n= 5 (20%)
